# Investigation on Electromigration-Induced Failure and Reservoir Effect in AlCu Interconnects

**DOI:** 10.3390/mi16040458

**Published:** 2025-04-13

**Authors:** Yuanxiang Zhang, Guoquan Jiang, Jingbo Zhao, Lihua Liang

**Affiliations:** 1 College of Mechanical Engineering, Quzhou University, Quzhou 324000, China; jgq@zjut.edu.cn (G.J.); 221124020461@zjut.edu.cn (J.Z.); 2 College of Mechanical Engineering, Zhejiang University of Technology, Hangzhou 310014, China

**Keywords:** aluminum–copper alloy, interconnects, electromigration, numerical simulation, reservoir effect

## Abstract

Aluminum–copper alloy (AlCu) is commonly utilized as interconnect material in low-power devices. However, as the size of electronic devices continues to decrease and current density increases, electromigration (EM) has emerged as a significant reliability concern for AlCu interconnects in the microelectronics industry. In this study, two-level AlCu interconnect structures with a Ti/TiN barrier layer were fabricated using 0.18 μm technology to perform accelerated EM tests. The test samples were subjected to three current levels (1.45 mA, 2.40 mA and 5.30 mA) at three ambient temperatures (200 °C, 225 °C and 250 °C) to investigate the nucleation and evolution of voids during EM degradation and then obtain the mean time to failure (MTTF). The numerical simulation method of atomic density integral (ADI) was used to simulate experimental observations based on the ANSYS platform, considering the coupled effects of electron wind force, stress gradient, temperature gradient, and atomic density gradient. A comparison of the experimental results and numerical simulations proves that the ADI method can be applied successfully to EM failure prediction of AlCu interconnects. Finite element models with different reservoir lengths were built to demonstrate the mechanism of the reservoir effect. The results show that the reservoir can improve the EM lifetime of the interconnect, but there is a critical extension length beyond which increasing extension sizes have no effect on EM lifetime.

## 1. Introduction

With Moore’s law slowing down, the advancement of integrated circuit (IC) packaging technology is accelerating. Emerging fields such as artificial intelligence (AI), 5G wireless technology, and new energy vehicles are propelling the evolution of advanced packaging towards three-dimensional, high-density, and heterogeneous integration [1]. In IC manufacturing, metallization interconnects serve as the primary conduits for signal transmission and act as critical bridges connecting unit circuits and linking multi-chip components within chip systems. In modern IC manufacturing, metallization interconnects have been reduced to micron or sub-micron scales [2], significantly increasing current density and making electromigration a crucial factor in IC reliability [3,4]. Electromigration is a mass transport phenomenon due to the momentum transfer between conducting electrons and metal atoms. This process typically leads to the formation and growth of voids in regions where atoms are depleted, thereby causing a substantial increase in electrical resistance and potentially resulting in metal line failure. Conversely, the accumulation of atoms can lead to the formation of hillocks, which may cause short circuits. The 2023 International Roadmap for Devices and Systems (IRDS 2023) emphasized that electromigration failure has become a primary limiting factor in the design of future large-scale and very-large-scale integrated (VLSI) circuits.

AlCu alloys have sustained their technological significance in the microelectronics industry for over five decades, following the discovery that copper doping substantially reduces electromigration degradation in aluminum interconnects [5]. Until now, AlCu continues to be extensively utilized in various VLSI devices that necessitate particularly low power consumption and cost-effectiveness, including analog-mixed signal and image sensor applications. For analog ICs, mature nodes such as 0.15 μm and 0.18 μm present advantageous options in terms of cost-effectiveness and performance. These nodes commonly employ AlCu metallization interconnects, and there is limited potential for further enhancement of electromigration resistance through process refinement. A good choice for delaying EM failure is to optimize the structure of interconnects, such as introducing an extension (also described as reservoir) at the end of interconnects. Many researchers have investigated the reservoir effect in AlCu interconnects with W vias [6,7,8]. However, the reservoir mechanism in AlCu interconnects and the impact of extension length on EM lifetime remain unclear.

The EM lifetime under normal device operating conditions is generally on the scale of several years. To achieve EM-induced failure within a reduced timeframe, accelerated electromigration tests are typically conducted at significantly elevated current densities and temperatures. The mean time to failure (MTTF) is predominantly utilized to characterize failure time and is defined as the point at which 50% of the test samples have failed. Black’s equation is widely used to evaluate the MTTF of interconnects for EM failure in the semiconductor industry [9]. Nonetheless, this empirical equation fails to provide a comprehensive understanding of the underlying physics associated with electromigration behavior, necessitating the development of more advanced and physically based models. It is generally accepted that electromigration is a multi-physics coupled field problem involved with electron wind, atomic density gradient (self-diffusion), stress gradient (stress migration) and temperature gradient (thermomigration) [10,11,12]. Many numerical models considering some or all of these diving mechanisms have been developed to predict the EM reliability of IC interconnects [13,14,15,16,17,18,19,20,21,22,23,24,25,26,27,28,29,30,31,32]. Blech initially proposed a steady-state solution that accounted for the equilibrium of atomic flux influenced by a stress gradient in opposition to the electrical current. [13]. Korhonen et al. [14] proposed a numerical model to derive the transient analytical solutions for hydrostatic stress and vacancy concentration distribution during electromigration without considering self-diffusion. Those EM models primarily focus on solving the partial differential equation (PDE) of hydrostatic stress evolution in confined multisegmented interconnects subject to blocking material boundary conditions. Recently, Chen et al. introduced a fast semi-analytic approach for combined electromigration (EM) and thermomigration (TM) analysis for general multisegmented interconnects. This new method was based on the separation of variables (SOVs) approach to find the analytic solution of coupled EM-TM PDE [17,18]. But all the analytical or semi-analytical EM models are used to obtain 1D numerical results.

Dalleau et al. [21], Weide-Zaage et al. [22] and Liu et al. [23] conducted a study on three-dimensional (3D) EM simulation employing the finite element method, which was based on the atomic flux divergence (AFD) and accounted for three driving forces: electron wind, temperature gradients and stress gradients. But Dandu [25] and Zhang [26] found that the AFD method would result in significant errors in predicting the void location of solder bumps because it neglected the effect of atomic density gradient. In our previous works [27,28], a more practical numerical simulation method of atomic density integral (ADI) was developed considering the coupled effects of electron wind force, temperature gradient, stress gradient, and atomic density gradient and was able to predict the EM failure of solder bumps and interconnects successfully. In addition, Cui [29,30] proposed a 3D-coupled EM theory that integrates a novel stress–strain constitutive equation. In this model, the strain induced by EM, as derived from the widely recognized stress-vacancy equation, is comprehensively integrated with other governing and field equations. Furthermore, the mass conservation equation, expressed in terms of total strain, is employed to characterize the diffusion process which is driven by the multi-physics of the electric field, temperature field, deformation field, and self-diffusion. Recently, a phase field model incorporating electrostatic free energy was developed to study the electromigration behavior of interconnects [31,32].

This paper aims to present a comprehensive electromigration study through experimental investigation and numerical simulation. First, EM test specimens were designed and fabricated using AlCu as the interconnect material. Second, EM accelerated tests were conducted under different ambient temperatures and current levels. The nucleation and evolution of voids, as well as the MTTF evaluation, were investigated according to the test results. Furthermore, a numerical simulation of EM was implemented to better understand the failure mechanism of EM. The numerical results were presented and compared with the experimental data, not only for void nucleation and evolution but also for EM failure time. The reservoir effect on EM lifetime in AlCu interconnects was investigated and discussed. This research will provide an optimized layout design for AlCu interconnects to enhance their resistance to EM failure, thus taking a step forward in IC design reliability.

## 2. Experimental Methods

**Figure 1:**
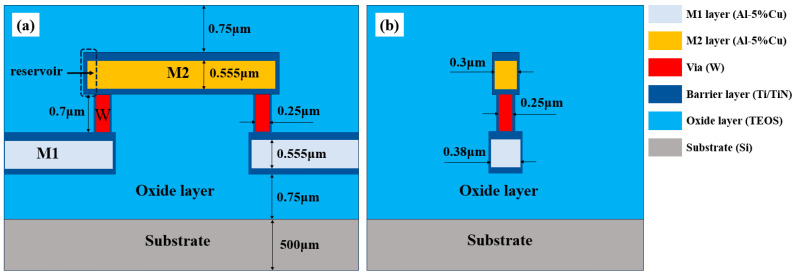
Schematic diagram of the EM test structure: (**a**) front view; (**b**) side view.

**Figure 2:**
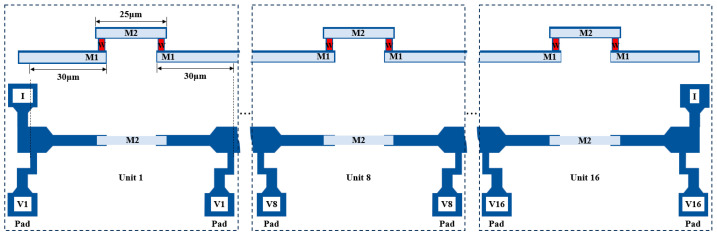
The EM test structure chain.

Two-level AlCu interconnect structures were fabricated utilizing 0.18 μm technology to investigate EM degradation, as shown in Figure 1. Both Metal line 1 (M1) and Metal line 2 (M2) layers were made of Al-5%Cu film. Tungsten (W)-filled vias were employed to establish an electrical connection between two metal layers. The tested metal lines of M1 and M2 had constant lengths of 30 μm and 25 μm, respectively, with single via of cross-sectional area 0.25 μm × 0.25 μm. Ti/TiN was deposited as a barrier layer to surround the whole metal lines. The thicknesses of Ti/TiN on the top and bottom of M1 and M2 were 0.075 μm and 0.05 μm, respectively. To investigate the impact of the reservoir on EM, the end of M2 structures were extended outward by 0.03 μm. The test structure was encapsulated in an oxide layer composed of tetraethoxysilane (TEOS) and utilized silicon (Si) as the substrate. To efficiently conduct the EM test, a test structure chain comprising 16 test units was designed, as illustrated in Figure 2. A pad structure was fabricated at every connection of the test unit in the M1 line to detect the voltage change of each unit during the EM test. The M1 lines connected to the pads were long and wide, which caused voids to be formed in the shorter and narrower M2 lines situated near the vias.

EM accelerated tests were conducted using a Qualitau package level EM test system (QualiTau, Inc., Santa Clara, CA, USA) at three ambient temperatures (200 °C, 225 °C and 250 °C) and three current levels (1.45 mA, 2.40 mA and 5.30 mA) to evaluate EM failure in a short time. The average current densities of the M2 line were 8.7 × 10^5^ A/cm^2^, 1.44 × 10^6^ A/cm^2^ and 3.18 × 10^6^ A/cm^2^, respectively. Before conducting EM accelerated testing, the test samples underwent annealing for 30 min at 400 °C in a forming gas atmosphere to stabilize their microstructure. A 10% resistance increase from the initial resistance of the metal lines was defined as the EM failure criterion. A minimum of 16 valid time-to-failure data points are required to obtain the MTTF for each case. The failure points of AlCu metal lines after the EM tests were observed using a scanning electron microscope (SEM).

## 3. Results and Discussions

### 3.1. Failure Analysis

**Figure 3:**
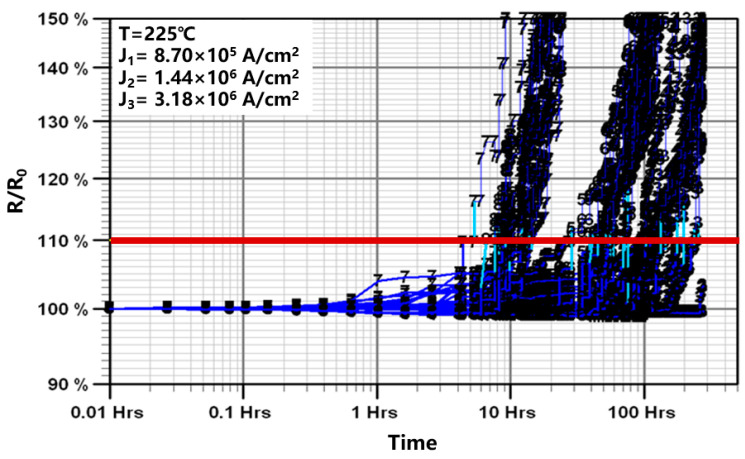
Resistance changes under accelerated stress conditions during EM tests at different current densities of 8.7 × 10^5^ A/cm^2^, 1.44 × 10^6^ A/cm^2^ and 3.18 × 10^6^ A/cm^2^ at 225 °C.

Figure 3 illustrates the resistance variation in AlCu metal lines subjected to EM testing under varying current conditions at a temperature of 225 °C. The resistance initially exhibited a gradual and slow change, which was subsequently followed by a sudden increase in resistance and a period of linear resistance rise. The initial period of gradual resistance change was attributed to the nucleation and growth of voids within the AlCu metal lines. This phenomenon occurred because EM-induced failure through void formation necessitates an adequate duration for both void nucleation and the continuous migration of atoms to facilitate void growth. As the voids expanded and became bigger, the interface resistance of the W via, Ti/TiN layer and AlCu metal line changed and resulted in a sudden increase in the resistance of the test structure. The final period of linear resistance rise was attributed to void growth beyond the W via so that the current had to pass through the thin and high-resistance Ti/TiN layer to connect the remaining AlCu metal line to the W via. It is important to note that EM is an irreversible process. As the current density or temperature increases, the EM failure becomes more severe, which increases the current density, causing more metal to migrate, creating damaging cycles.

**Figure 4:**
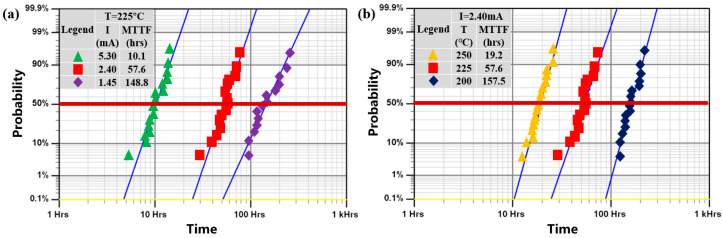
Cumulative EM failure probability plots at different accelerated conditions. (**a**) At 225 °C; (**b**) at 2.40 mA current.

The cumulative EM failure probability distribution of MTTF is shown in Figure 4. The distributions are described well with log-normal distributions with similar slopes, indicating similar failure modes at these accelerated conditions. Specifically, higher current density and temperature will accelerate the growth of voids, resulting in a shorter MTTF. The effect of changes in current density and ambient temperature on the EM lifetime of the interconnect can also be verified using Black’s equation.

**Figure 5:**
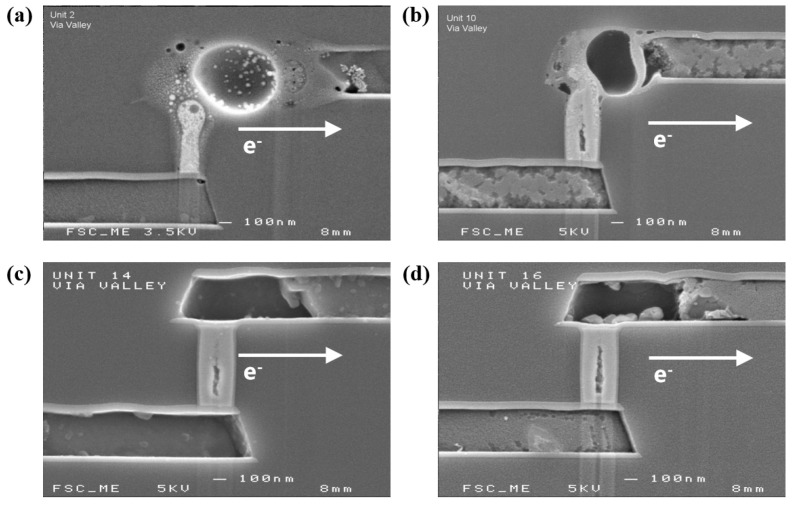
SEM image of several typical test units after accelerated EM test. (**a**) Unit 2; (**b**) unit 10; (**c**) unit 14; (**d**) unit 16.

Figure 5 presents the cross-sectional scanning electron microscopy (SEM) image of several representative test units following an accelerated electromigration (EM) test conducted at 225 °C with a current of 1.45 mA. It can be clearly observed that continuity voids were formed at the end of the M2 for both unit 14 and unit 16. Throughout the EM process, voids first nucleated along the interface of Ti/TiN/M2 near the vias and progressively expanded to form extensive voids at the cathode of the M2 layer, ultimately resulting in EM-induced failure of the interconnect. The reservoir at the end of the M2 was also involved in the electromigration process. In fact, the layout with a reservoir can improve the failure time of M2 by providing additional metal ions when metal ions move from the cathode to the anode during EM.

### 3.2. Finite Element Model

**Figure 6:**
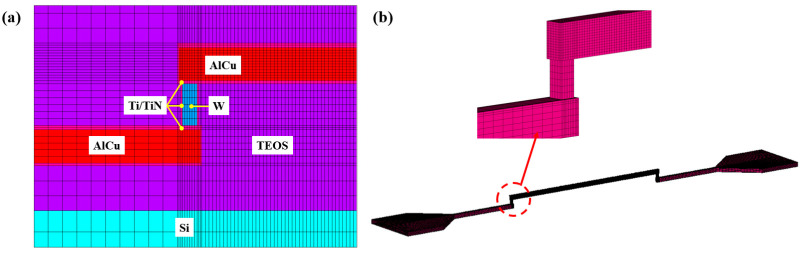
Finite element model of the investigated test unit. (**a**) Mesh of the whole model; (**b**) mesh of the AlCu and Ti/TiN layer.

A three-dimensional finite element model was built in ANSYS software (ANSYS 2022 R1) to further understand the EM failure characteristics observed in the experimental studies. Due to the periodic symmetry of the test structure, only one test unit was built. The finite element model was discretized using hexagonal elements, as illustrated in Figure 6. According to the accelerated EM test conditions, three different current loads of 1.45 mA, 2.4 mA and 5.3 mA were individually applied at the end of the pad. The ambient temperature surrounding the test structure was 200 °C, 225 °C and 250 °C, respectively. Electrical, thermal and mechanical material properties for simulation were taken from the literature [23,24,33].

**Figure 7:**
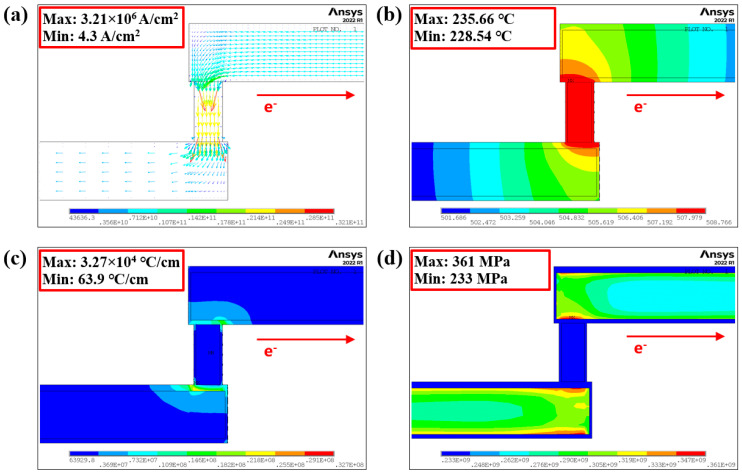
Finite element analysis results with 1.45mA current applied at ambient temperature of 225 °C. (**a**) Current density; (**b**) temperature; (**c**) temperature gradient; (**d**) hydrostatic stress.

Figure 7 shows the distributions of current density, temperature, temperature gradient, and hydrostatic stress within the test unit under ambient conditions of 225 °C and an applied current of 1.45 mA. The maximum magnitude of current density in the M2 line is 3.21 × 10^6^ A/cm^2^ at the location where electrons enter the M2 line from the via. This value is four times greater than the average current density in the bulk of the M2 line (8.7 × 10^5^ A/cm^2^). Current crowding, which arises from abrupt changes in cross-sectional area, is the primary factor contributing to electromigration. The interface between the Ti/TiN layer and the M2 line exhibits a maximum temperature of 235.66 °C, attributed to the increased difficulty in dissipating heat at this interface compared to other locations. The maximum temperature gradient is calculated as 3.27 × 10^4^ °C/cm. Tu identified a threshold temperature gradient of 1000 °C/cm as necessary to consider the impact of the temperature gradient [34]. Due to the significant temperature gradient, the effect of thermomigration cannot be ignored. In addition, the hydrostatic stress is also relatively higher at the Ti/TiN/M2 interface at the cathode of M2. Therefore, it is easy to nucleate voids at the Ti/TiN/M2 interface, consistent with the experimental results. In fact, effective thermal management is critical to the reliability of interconnects. Many researchers are employing thermal-enhancing technologies such as thermal interface materials and advanced thermal solutions to ensure that interconnects are not subjected to excessive thermal stress and to minimize the potential for EM.

### 3.3. Numerical Simulation of Electromigration

Electromigration is a diffusion process governed by the mass continuity equation. The evolution of the local atomic density within the interconnect can be described by the following equation:

(1) $$\frac{\partial c}{\partial t}+\nabla \cdot q=0$$

where c is the normalized atomic density, and q is the total atomic flux of normalized migration caused by various driving mechanisms.

As mentioned before, the driving mechanisms of atomic flux include electron wind, atomic density gradient, stress gradient and temperature gradient. Thus,

(2) $$q=q_{\mathrm{Ew}}+q_{\mathrm{Th}}+q_{S}+q_{C}=\frac{\mathrm{cD}}{k_{B}T}eZ^{*}j\rho -\frac{\mathrm{cD}}{k_{B}T}Q^{*}\frac{\nabla T}{T}-\frac{\mathrm{cD}}{k_{B}T}\Omega \nabla \sigma_{H}-D\nabla c$$

where $D=D_{0}exp\left(-E_{A}/k_{B}T\right)$ is the effective diffusion rate, $D_{0}$ is the initial diffusion coefficient, $k_{B}$ is the Boltzmann constant, $E_{A}$ is the activation energy, T is the absolute temperature, j is the current density vector, e is the electronic charge, $Z^{*}$ is the effective charge number, ρ is the temperature-dependent resistivity, $Q^{*}$ is the thermal conductivity, Ω is the atomic volume of AlCu metal, and $\sigma_{H}$ is the hydrostatic stress.

Because the AlCu metal line is surrounded by a barrier layer of Ti/TiN, there is no atomic flow at the boundary of AlCu. The blocking boundary condition for atomic flux is applied to the EM evolution equation (Equation (1)) within a closed domain V on boundary Γ.

(3) $$q\cdot n=0\begin{array}{cccc} \; & \mathrm{on} & \; & \Gamma \end{array}$$

The normalized atomic density c determines the void formation. Initially, the normalized atomic density is set to $c_{0}=1$. Equation 1 with specified boundary and initial conditions describes the evolution of atomic density at any location within the interconnect segment characterized by the given j, T, ∇T and $\nabla \sigma_{H}$. We have developed the ADI algorithm based on the discretized weighted residual method (WRM) to solve Equation (1) using the variable of atomic density. Initially, the distributions of current density, temperature and stress within the interconnect are determined through finite element analysis in ANSYS. Subsequently, atom density redistribution in the considered interconnect is addressed using a user-defined FORTRAN code. To determine the formation of dynamic voids, finite elements with the lowest normalized atomic density are selectively removed from the model. Around the removed elements, a void begins to form and expand. This process is followed by a series of simulations that include the analysis of the modified model, the calculation of normalized atomic density, and the deletion of the least stressed elements, continuing until the structure ultimately fails. The detailed ADI method and its computation procedure can be found in our previous works [23,24].

**Figure 8:**
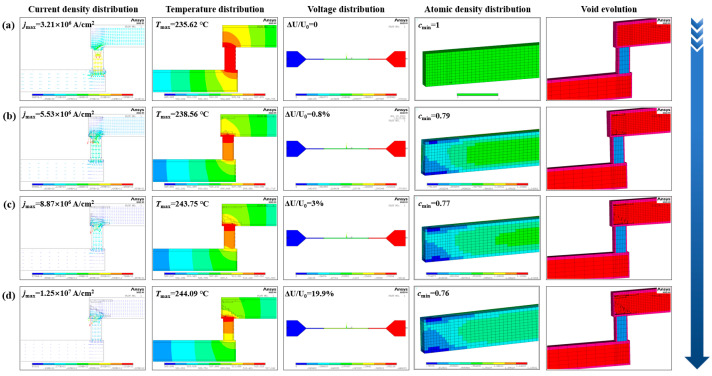
Dynamic evolution simulation of current density, temperature, voltage atomic density and voids at different calculated times under a constant current of 1.45mA at an ambient temperature of 225 °C. (**a**) At initial time; (**b**) at the calculated time of 118.8 h; (**c**) at the calculated time of 134.5 h; (**d**) at the calculated time of 135.6 h.

**Figure 9:**
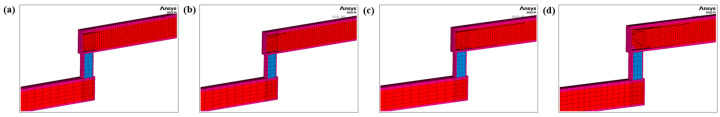
Void formations for EM failure under different conditions. (**a**) 2.4mA at 200 °C; (**b**) 2.4mA at 225 °C; (**c**) 2.4mA at 250 °C; (**d**) 5.3mA at 225 °C.

Figure 8 illustrates the evolution of current density, temperature, voltage, atomic density and void formation within the test structure at different calculated times under a constant current of 1.45mA at an ambient temperature of 225 °C. The minimum atomic density was observed at the cathode of the M2 line, specifically near the M2 and Ti/TiN interface. This result indicates the start of the depletion process at this location, which was consistent with the experimental findings. For the void evolution simulation, a set of 50 elements was chosen for deletion per step, requiring more than three steps to complete the simulation. During void formation, the cross-sectional area of the M2 line at this location decreased, resulting in a significant increase in current density and a subsequent local temperature rise due to joule heating. According to Figure 8, the total time-to-failure (TTF) for EM was determined to be 135.6 h based on a three-step calculation of time. During the initial step, the M2 line required 118.8 h to initiate void formation, indicating that the void generation occupies most of the EM failure time. As the void continued to expand, there was a rapid increase in current density, stress gradient, and temperature gradient within the void region, resulting in a significant reduction in the calculated failure times for the second and third steps, which were 15.7 h and 1.1 h, respectively. Furthermore, during the first and second stages of void evolution, changes in voltage were negligible because the void occupied only a portion of the M2 cross-section. However, in the third step, as the void extended across the entire M2 cross-section, the current was forced to flow through the higher-resistance Ti/TiN layer, causing a sharp voltage increase and a 19.9% jump in resistance. In addition, analogous void formations in the M2 line are observed in cases of electromigration failure under various other conditions, as illustrated in Figure 9.

**Figure 10:**
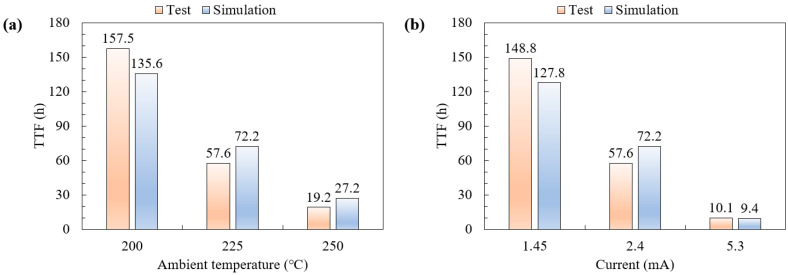
Comparison of the simulation and experimental test results for TTF. (**a**) Different ambient temperatures at 2.40mA; (**b**) different currents at 225 °C.

A comparison of the simulation and experimental test results for TTF is presented in Figure 10. It indicates that the simulated TTF data agree well with the tested MTTF. The increase in both ambient temperature and current will accelerate the EM failure of the interconnect. Due to the process defects in the actual test structure compared to the finite element model, there is little difference in TTF between the simulated and experimental test results.

### 3.4. Effect of Reservoir on Electromigration

**Figure 11:**
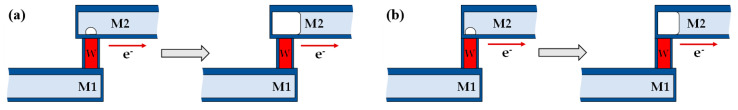
Schematic diagram of the effect of the reservoir on the EM failure mechanism. (**a**) With reservoir; (**b**) without reservoir.

Figure 11 shows the effect of the reservoir on the EM failure mechanism. For the interconnect structure, a reservoir (as shown in Figure 11a) acts as a supplement of metal atoms. When metal atoms from the M2 layer above the via move from the cathode to the anode during EM, metal atoms in the reservoir will replenish them to slow down the formation and growth of voids, thus improving the EM reliability of the interconnect. For the interconnect structure without reservoir (as shown in Figure 11b), the failure time is shorter because there are no additional metal atoms to fill the voids during EM. According to this mechanism, a reservoir is generally required at the end of the interconnect.

**Figure 12:**
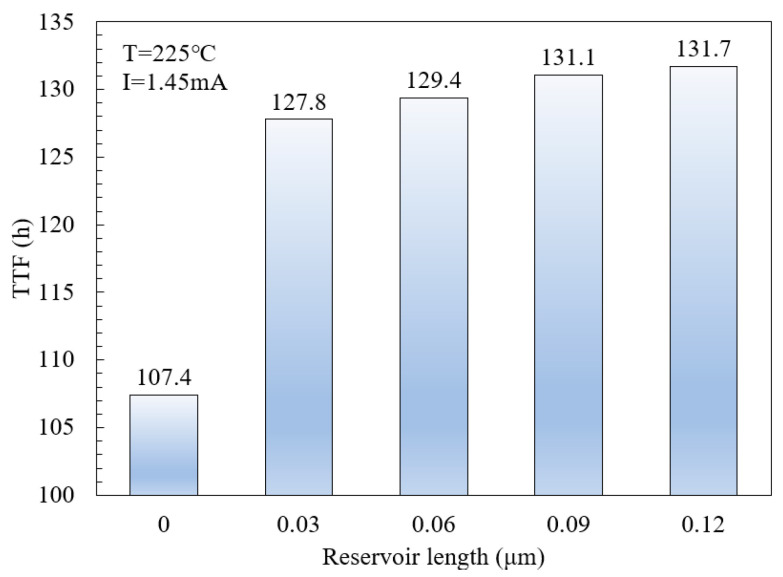
Effect of reservoir length on TTF under a constant current of 1.45mA at an ambient temperature of 225 °C.

To further understand the reservoir length effect on the interconnect, a numerical simulation of electromigration with different reservoir lengths was performed and the different TTF results are shown in Figure 12. It can be seen that the EM lifetime of the interconnect structure with a reservoir is larger than the one without a reservoir. Specifically, as the reservoir length extends from 0 to 0.03 μm, the TTF escalates from 107.4 h to 127.8 h, reflecting an enhancement in EM lifetime of approximately 19%. However, an increase in reservoir length from 0.09 μm to 0.12 μm does not yield a substantial improvement in EM lifetime. Previous studies have identified a critical reservoir length beyond which further extensions do not prolong EM lifetimes [7]. The current analysis suggests that a reservoir length of approximately 0.09 μm was predicted as the critical length, beyond which additional extensions do not influence EM lifetimes under the high current density and temperature conditions of this study.

## 4. Conclusions

In this paper, a comprehensive study of EM for AlCu interconnects driven by electron wind, atomic density gradient, stress gradient and temperature gradient has been conducted through experimental and numerical simulations. The reservoir mechanism in the AlCu interconnect and the impact of extension lengths on EM lifetimes were also investigated. The main conclusions of this study are summarized below:

1. AlCu interconnects have similar failure modes at high current densities from 8.7 × 10^5^ A/cm^2^ to 3.18 × 10^6^ A/cm^2^ and high temperatures from 200 °C to 250 °C, according to the EM accelerated tests. A higher current density and temperature will accelerate the growth of voids, resulting in shorter EM failure times. Voids first nucleated along the interface of Ti/TiN/M2 near the vias and gradually expanded to form extensive voids at the cathode of the M2 layer, which was verified by both experimental and simulation results.
2. Reliability modeling and simulation tools are being used to better understand the EM reliability of interconnects. This proactive approach can help us to identify potential problems before they arise and then take targeted preventive measures. The ADI method introduced in this paper has been successfully performed to predict the EM failure of AlCu interconnects and the simulation results for void formation and TTF have reasonably good correlation with the test results. The temperature gradient, which exceeds 1000 °C/cm cannot be ignored when the AlCu interconnect is under stress at a high current density and temperature.
3. A reservoir will improve the EM failure of AlCu interconnects effectively. However, only a portion of the extended volume can function as an effective reservoir for void accumulation. Under the high current density and temperature conditions of this study, an extension of approximately 0.09 μm was predicted as the critical length, beyond which further increases in extension size do not impact EM lifetimes.

## Data Availability

Data are available on request.
